# A Conservative Management Approach for Unusual Presentation of Oral Actinomycosis

**DOI:** 10.1155/2021/5570758

**Published:** 2021-05-18

**Authors:** Qamar Hashem

**Affiliations:** Clinical Dental Sciences Department/Endodontic Division College of Dentistry, Princes Noura Bint Abdulrahman University, Riyadh, Saudi Arabia

## Abstract

Actinomycosis is gram-positive saprophytic infection that is characterized by chronic suppurative and granulomatous lesion. It could be found in the oral cavity, lungs, colon, and genital area. In the oral cavity, it is commonly associated with infected root canals presented as persistent infections. This case reports demonstrate an atypical presentation of actinomycosis in the lower left mandibular canine/premolar area showing painless soft tissue lesion associated with bone sequestration. Nonsurgical curettage of the lesion followed by nonsurgical root canal treatment and retreatment to the offended teeth was determined as the treatment modality for this case.

## 1. Introduction

Actinomycosis is saprophytic infection that is characterized by chronic suppurative and granulomatous lesion by endogenous flora of the mouth [1]. Actinomycosis is caused by microaerophilic or anaerobic types of bacteria that usually colonize in the oral cavity and other areas of the body like the lungs, the female genital tract, and the gastrointestinal tract [2]. It belongs to the nonacid fast gram-positive bacteria, and most of the species identified from the actinomycosis lesion are *Actinomymyces israelii*, *Actinomymyces odontolyticus*, *Actinomymyces viscosus*, *Actinomymyces naeslundii*, or *Actinomymyces meyeri* [3].

Intraorally, the infection could present in chronic, acute, or subacute forms. Involving both the bone and soft tissue together or one of them solely, the infection spreads in atypical form in to the surrounding organs or tissues leading for sinus tracked production [4].

Cervicofacial actinomycosis rarely involves the bone; nevertheless, it affects the soft tissues more frequently. The incident differs depending on the location, the temporomandibular joint, chin, cheek, and the maxilla that are the less common affected locations. However, more than 50% of the cases are found in the mandible [5].

Periapical actinomycosis is believed to be a nonresolving periapical lesion associated with actinomycotic infection and has been suggested as a contributing factor in the perpetuation of periapical radiolucencies after root canal treatment. Actinomycotic osteomyelitis of the mandible is an uncommon consequence of odontogenic infections [6]. Diagnosis is especially difficult and usually made by identifying the typical actinomycotic colonies in a surgical specimen [4]. Periapical actinomycosis is thought to be rare, and the data reported the frequency of periapical actinomycosis and the possibilities of it that is correlation between such condition and cervicofacial actinomycosis is limited [7].

Typically, a disruption in the integrity of the mucous membrane due to injury, infection, or surgical intervention may allow microorganisms to access deeper structure and disease progression. In some conditions, microorganisms emerging from the canal can conquer the protective barriers and produce an extraradicular contamination, thus giving the clinical presentation of a periapical lesion with a bone fragment of osteomyelitis in the mandible [6].

In this case report, we are presenting a lesion in the mandibular left side related to a poor endodontically treated tooth having both a periapical lesion and a segment of the necrotic bone.

## 2. Case Presentation

A 56-year-old male complains from a white patch on his facial gingiva of his lower teeth that causes him discomfort and pain that started few weeks ago. The patient with no knowing allergies reported a medical history of myocardial infarction two years ago, hypothyroidism and dyslipedimia. In addition, he is a heavy smoker for more than 20 years. As a result, he is under multiple medications in order to manage his general health like bisoprolol fumarate (Concor), perindopril arginine (Coversyl), and clopidogrel bisulfate (Plavix).

The patient reported localizes swelling in the lower left area few months ago. The patient was giving an antibiotic course and pain medication by their local doctors and referred the patients to the ENT. Then, nystatin was prescribed as antifungal medication, but no improvement was noted, and the complaint persists. Following that, the patient was referred to the dental department for proper management and long-term planning.

**Figure 1:**
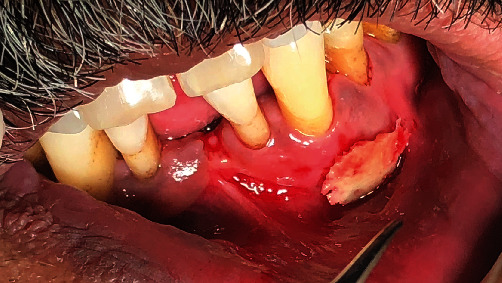
Intraoral picture of lesion in buccal vestibule of the mandibular left side.

The intraoral examination revealed a soft tissue lesion associated with bone sequestration on buccal mucosa relative to tooth number #33 and #34 (Figure 1).

Tooth #33 is sound, but tooth #34 had previous root canal treatment (RCT) with apical transportation and was restored with temporary filling few years ago. However, no pus discharged or any lymphadenopathy was noted. The general practitioner few weeks before endodontic appointment did a sinus tract tracing using gutta-percha point size 30.

**Figure 2:**
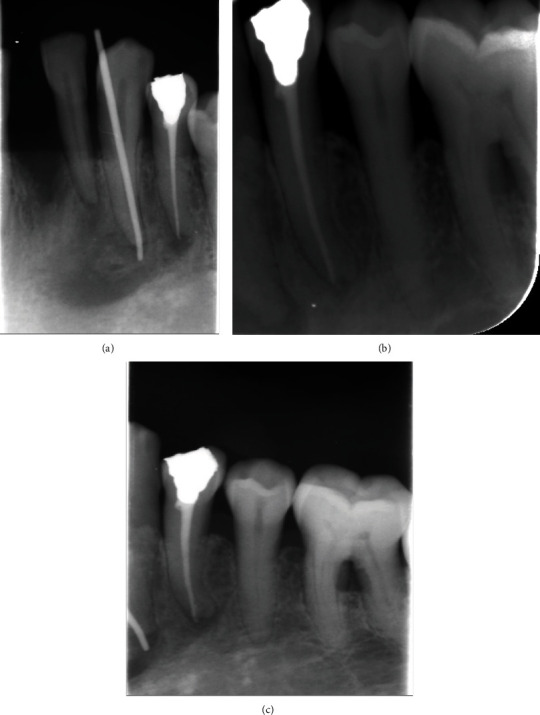
Intraoral preoperative periapical radiograph showing an irregular periapical radiolucency involving both #33 and #34. (a) Tracing was made using gutta-percha point done. (b, c) Other periapical angles.

**Figure 3:**
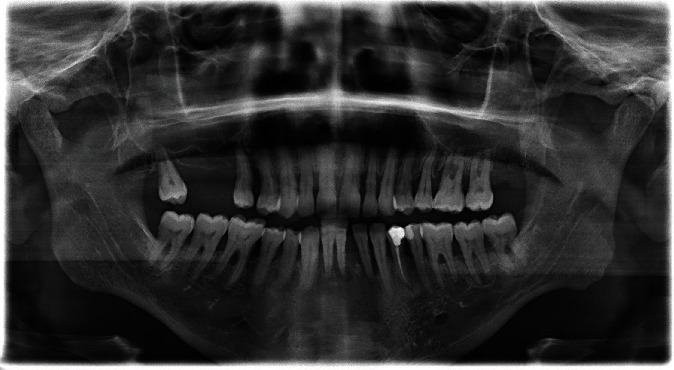
Orthopantomogram showing multiple missing teeth and endodontic treatment of tooth #34.

A provisional diagnosis of chronic infection lesion made based of the provided history and clinical findings. These finding were more investigated through radiographical examination, an orthopantomogram (OPG) (Figure 3) and periapical radiograph for tooth #34 (Figure 2) were taken. Finding includes generalized periodontitis, and missing teeth #26 and #27 and periapical radiolucency extended from tooth #32 to tooth #34.

**Figure 4:**
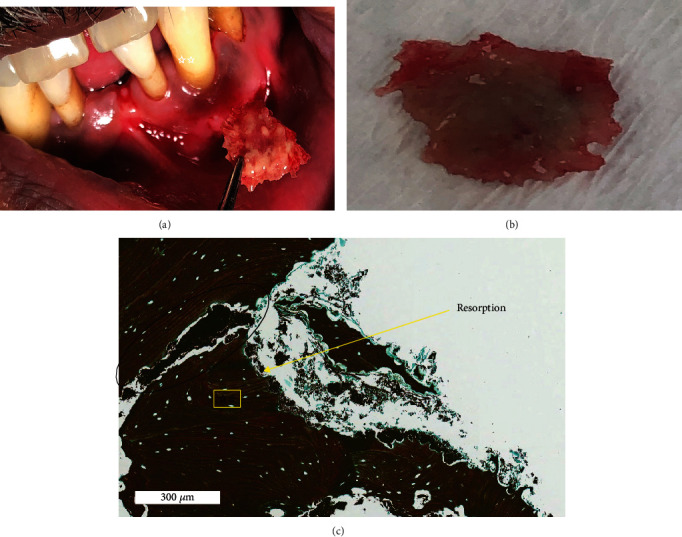
(a, b) Intraoral photograph after bone removal. (c) Necrotic bony trabeculae with actinomycosis filaments on the trabecular surface (black circle) and extensive sclerosis of bone showing prominent resting and reversal lines (^∗∗^).

In order to exclude malignancies as a differential diagnosis, routine blood examination, excisional biopsy, and culture sample from the lesion were performed. Regarding the routine blood test, apart from elevated neutrophils and lymphocytes, no abnormalities were detected. The pathological report of the biopsy revealed fragments of the necrotic bone and bacteria. In addition, trabecular surface and bone sequestrum of the necrotic bone were showing actinomycosis colonies (Figure 4). Therefore, a definitive diagnosis of actinomycosis was made. No histological sampling of the periapical root structure was performed at the time, as was not seen as necessary since the root structure has been curettage with the surrounding area.

The treatment plan will include patient education and motivation, nonsurgical curettage of the lesion, and nonsurgical root canal retreatment for tooth #34. Prior to removal of lesion, vitality testing of the adjacent teeth (#32, #33, #35, and #36) was assessed and gives vital normal response except for tooth # 33 which gives negative response. Accordingly, RCT for tooth #33 added to the treatment plan.

**Figure 5:**
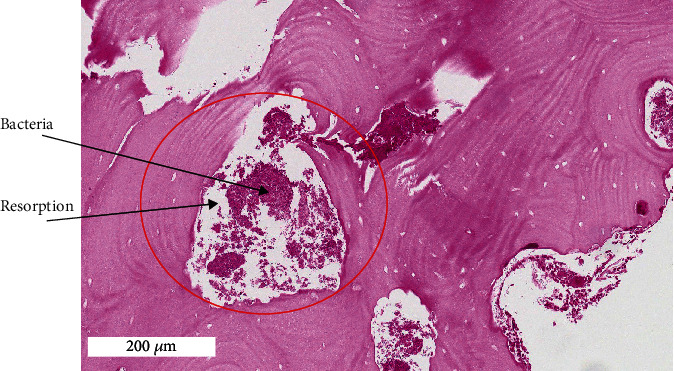
H&E stain showing bacterial invasion in the bone causing resorption. Bacteria (black arrow) and resorption of bone (black stars).

Removal of the bony sequestration (0.5 × 0.5 × 0.15 cm) and placed in formalin was carried out and submitted for histopathological analysis as well as swap from the exposed area (Figure 5).

**Figure 6:**
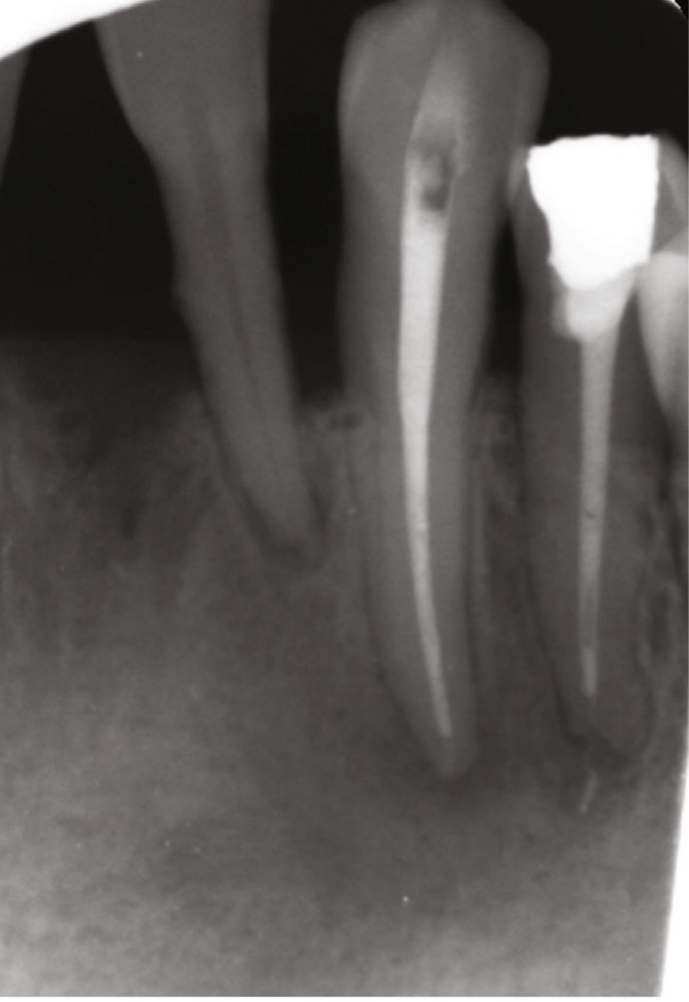
Postoperative periapical radiograph showing endodontic treatment of both teeth #33 and #34.

Lastly, curettage of the area and irrigation with saline done, augmentin 1 g twice/day (bid) for one week, was prescribed and patient schedule for treatment. In the subsequent visit, local anesthesia (LA) as xylocaine with epinephrine 2% administered a rubber dam (RD) isolation for both #33 and #34. Tooth #34 previous root canal filling was removed and access done, working length determination, cleaning and shaping done using the rotary system (crown down technique), and 5.25% NaOCl used for irrigation. Due to previous treatment misshape of an open apex and failure to achieve apical stop, mineral trioxide aggregate (MTA) plug placed at the apical 1/3. Obturation of the remaining canal with gutta-percha and AH 26 sealer, with continuous wave compaction, temporized with cavit and silver glass ionomer (Ketac) as temporary (Figure 6). Tooth #33, a conventional RCT, carried out following the same steps of tooth #34 but obturation was made using gutta-percha and AH 26 sealer (Figure 6), and then the tooth was restored with temporary filling. The slight amount of extruded MTA will not affect the healing due to its compatibility [8].

**Figure 7:**
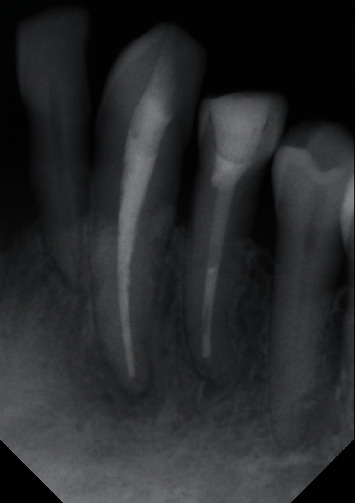
1-year recall periapical radiograph showing healing of the lesion.

Patient referred for periodontist in order to manage the generalize periodontitis and then for prosthodontist for final restorations for #33 and #34. The patient was followed up at six months and one year period with no evidence of lesion recurrence (Figure 7).

## 3. Discussion

Actinomycosis is one of the infectious diseases that affect both humans and animals in a chronic, granulomatous form. The contribution factors for endodontic treatment failures are many; however, with it is low prevalence among all periapical lesions (less than 5%), actinomycosis cannot be consider as a common cause leading for RCT failure. Cervicofacial actinomycosis is classified into two subtypes, peripheral and central [7]. While the central type is considered very rare, actinomycosis falls under central type which itself is very uncommon among central varieties [9]. Early diagnosis of actinomycosis combined with proper intervention has an excellent prognosis. Cure rates are high, and neither deformity nor death is common.

Knowing that extraradicular infection can be dependent or independent of the intraradicular infections, *Actinomyces* and *Propionibacterium propionicu* species are the only species capable of forming an independent extraradicular infection in pathological entity named apical actinomycosis. In this case, the endodontic failure may be due to both the previous substandard endodontic treatment and the presence of the *Actinomyces* species in the periapical area [10, 11].

Many local (oral) and general health factors contribute to the physiopathological pathways of cervicofacial actinomycosis. Poor oral hygiene, dental caries, gingival inflammation or trauma, traumatic dental extraction, and cervicofacial surgery are among the oral factors. On the other hand, general health factors include immunocompromise and diabetic patients, male gender, and steroids [12]. In our presented case, the patient had many of these risk factors like age, medical conditions, and poor oral health.

Most of the publications on apical actinomycosis are case reports. Many treatment approaches were done that depend on the case. Alhezami in 2010 did incision and drainage of the lesion. Two weeks later, endodontic apical surgery was done [13]. On the other hand, extraction of the involved teeth in order to evaluate the deeper tissue and relief the pain considered to manage actinmycosis. This was done by Gannepalli et al., and few teeth were extracted followed by debridement of the area. In our case, the infection pathway was extremely irregular leading for osteomyelitis and hard texture alongside free gingiva and buccal vestibule location [5]. As a result, a conservative surgical approach by excisional biopsy and root canal retreatment was ending to symptom relief and proper healing. In addition, avoiding extraction when possible will lead for better oral health and improve the patient quality of life.

MTA used apically for 5 mm for RCT retreatment for #34. A 5 mm barrier demonstrated significantly greater microhardness than the 2 mm barrier MTA used after root canal preparation favored the occurrence of the apexification and periapical healing. The initial use of Ca (OH)_2_ paste was not necessary for apexification to occur and has shown to be strongly related to the extrusion of MTA and formation of barriers beyond the limits of the root canal walls [14]. Low cytotoxicity and the induction of mineralization are 2 desirable properties of MTA that may explain the bioregenerative and the biocompatibility of this biomaterial. The extrusion of the MTA root canal filling biomaterial from an open apex tooth into a periradicular lesion does not produce any complications [15].

Apart from the periapical type of actinomycosis, long-term systemic antibiotic therapy has been the treatment of choice for all the other types of actinomycosis. With duration ranging usually from 3 months to 1 year, the penicillin is the first drug of choice, and erythromycin and tetracyclines are alternative options [16]. In long-standing infections, fibrosis can reach in to advance level of sclerosis that inhibits the antibiotic penetration as a result of the poor vascularity. In addition, the sulfur granule has a protective nature, making high doses needed for effective therapy following RCT, extraction, or surgical intervention [16]. In our case, the previous oral antibiotic taken by the patient was ineffective. However, after lesion removal and RCT, antibiotic was prescribed as shown by some reported cases and considering patient medical history.

## 4. Conclusion

Oral periapical actinomycosis is a long-standing infection presents in unusual ways in the mouth. With its benign potential, educating the health care providers and general dental practitioner will result in better transfer of the case for proper diagnosis and management. Using cultures and histopathology to reach the definitive diagnosis, a conservative approach that avoids extraction should be considered if possible specially when it reliefs the patient symptoms and provides good prognosis.

## Data Availability

Data available are in the main text.
